# Effect of Pea Protein Isolate–Soybean Meal Ratio on Fiber Structure and Texture Properties of High-Moisture Meat Analogs

**DOI:** 10.3390/foods13233818

**Published:** 2024-11-27

**Authors:** Zhongjiang Wang, Yachao Tian, Fangxiao Lou, Zengwang Guo

**Affiliations:** College of Food Science, Northeast Agricultural University, Harbin 150030, China

**Keywords:** pea protein isolate, soybean meal, meat analogs, textural properties, scanning electron microscopy

## Abstract

Inadequate fibrous attributes and prohibitive pricing are pivotal barriers to the broader market penetration of meat analogs (MAs). This research endeavors to address these impediments by formulating a blend of cost-effective soybean meal (SM) and pea protein isolate (PPI) across a spectrum of ratios (PPI:SM = 1:0, 8:2, 6:4, 4:6, 2:8, and 0:1). The analysis of textural properties elucidated that the integration of SM markedly diminished the textural rigidity and mastication resistance of MAs. Employing scanning electron microscopy (SEM) and fibrillation degree metrics, it was ascertained that the most favorable fibrous architecture of MAs was attained at a PPI to SM ratio of 6:4. Further experimental evidence underscored that the synergistic interaction between SM and PPI catalyzed the conversion of free sulfhydryl groups into disulfide linkages, a pivotal mechanism in the augmentation of MAs’ fibrous matrices. The conclusions drawn from this study provide substantive contributions to the formulation of superior-quality, economically viable MAs, and could potentially accelerate their market acceptance.

## 1. Introduction

With the growth of the global population, the demand for meat as a food source continued to rise [1,2]. However, traditional methods of animal meat production not only consumed a large amount of resources, but also had a serious impact on the environment [3]. In addition, the high cholesterol and fat content of animal meat, along with potential food safety issues, attracted widespread attention [4]. Therefore, developing healthier, environmentally friendly, and sustainable meat substitutes became the focus of current research. As a new meat substitute, with vegetable protein serving as the main raw material, plant-based meat analogs (MAs) could imitate the fiber structure of meat [5]. Extrusion was the most effective feasible technology for processing plant-based MAs [6]. Products produced through low-moisture extrusion typically required rehydration before consumption and further processing, and their texture tended to be relatively rough [7]. High-moisture extrusion technology could significantly enhance the chewing sensation and elasticity of MAs, aiding in retaining nutrients within MAs and promoting a uniform distribution of nutrients. This improved the overall nutritional value of MAs, making them closer to the eating experience of traditional animal meat [8].

The plant proteins utilized in the production of MAs have been primarily sourced from soybeans [9,10], peas [11,12], and wheat [12,13]. Pea protein isolate (PPI) garnered increasing attention due to its advantages, such as its nutritional value, digestibility, non-toxicity, and non-transgenic origins [14,15]. However, it was found that the texture of MAs prepared by PPI through high-moisture extrusion was poor, and the fiber structure was not similar to that of animal meat [12]. It was found that MAs produced with protein from two different sources under high-moisture conditions exhibited a superior fiber structure. For instance, the combination of soybean protein isolate (SPI) and wheat gluten protein (WG) could effectively enhance the structural and textural properties of MAs [16]. Based on this observation, we believed that the fiber structure and quality of MAs could be effectively improved through high-moisture extrusion when PPI was compounded with other types of protein. In fact, the high cost was an important factor limiting the promotion of MAs. This was primarily due to the relatively high prices of SPI, PPI, and WG used in the manufacturing of MAs. Soybean meal (SM), another prevalent source of plant protein, is abundant in amino acids and nutrients, and its price is significantly lower than that of SPI, PPI, and WG due to the simplicity of its process. Soybean meal (SM) represents the most basic form of soybean protein [17]. The allergenicity of PPI is low, and its allergens primarily originate from two types of globulins, Pis s1 and Pis s2 [18]. SM is the main byproduct of soybean processing, and its allergenic proteins primarily include β-conglycinin and glycinin [19]. Although PPI and SM contain some allergenic substances, the research shows that high-temperature and high-pressure treatment in the process of high-moisture extrusion can effectively reduce allergenicity [20]. Through previous experiments, we found that MAs prepared by SM alone have almost no fiber structure, which obviously cannot meet the requirements of the market and consumers for MA quality. The combination of PPI and SM can not only reduce the cost, but also further improve the texture and fiber structure of MAs through their complementary effects. However, plant proteins may have different physical and chemical properties and interaction mechanisms. Therefore, it is necessary to systematically study the influence of the ratio of PPI to SM on the texture and fiber structure of MAs during high-moisture extrusion.

In this study, PPI and SM were mixed in different proportions (PPI:SM = 1:0, 8:2, 6:4, 4:6, 2:8, and 0:1). MAs were produced through a high-moisture extrusion process. The texture properties of MAs were measured by a texture analyzer. The changes in the fiber structure of MA samples was observed by a scanning electron microscope (SEM). In order to deeply understand the interaction between PPI and SM, the secondary structure of MA samples was analyzed. We assessed the textural characteristics of MAs using a texture analyzer. SEM imaging was employed to observe alterations in the fiber structure of MA samples. In addition, in order to deeply understand the interaction between PPI and SM, we detected the number of sulfhydryl groups and disulfide bonds in MAs. The research results can be applied to the development of new MA foods and health products.

## 2. Materials and Methods

### 2.1. Materials

The PPI and SM samples were sourced from Yuwang Group (Yucheng City, Shandong Province, China). The preparation method of PPI was as follows: Soak peas in water for about 20 h, and then grind them with a grinding wheel. Bean dregs were repeatedly ground 3 times, and the residue was removed. Add acid to the supernatant to adjust the pH to about 4.5 to precipitate protein, add alkali to adjust the pH of protein after acid precipitation to about 7.2 to dissolve protein, and then homogenize and spray dry to obtain PPI. PPI comprises approximately 85% protein content, with 12.5% carbohydrates, 1% fat, and a moisture level of 3%. The preparation method of SM was as follows: clean soybean, soak it in water, and cook to inactivate lipase. Then, the crude soybean meal was obtained by low-temperature pressing to remove oil and part of the water. Then, the squeezed SM was dried at a low temperature to further reduce the moisture content and obtain SM. SM has a composition of roughly 57% protein, 20.5% carbohydrates, 2.5% fat, and 7% moisture. Additional chemicals and reagents were supplied by Yuanye Biotechnology Co., Ltd. (Shanghai, China).

### 2.2. MA Preparation

**Figure 1:**
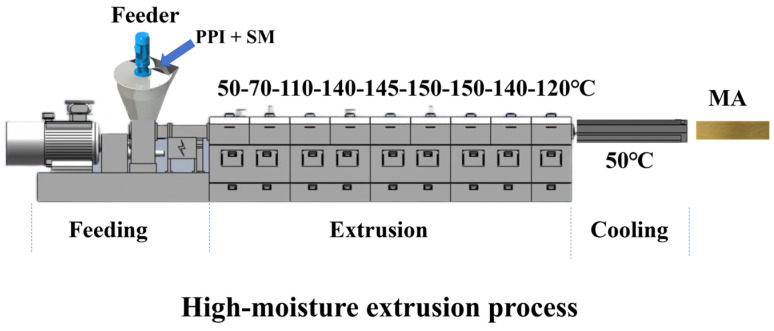
Schematic diagram of the extruder.

MAs were prepared using an HT-36 extruder (Shandong Arrow Machinery Co., Ltd., Jinan, China). PPI and SM were fully mixed in a WLDH-500 mixer (Jiangsu Hongda Powder Equipment Co., Ltd., Jiangyin, China) to prepare the feed mixture (PPI:SM = 1:0, 8:2, 6:4, 4:6, 2:8, and 0:1). The extruder barrel comprised ten sections. The first section included the gate for solid feed, while the tenth section featured individual temperature controls. The extruder parameters were set as follows: the feeding speed of the PPI and SM mixture was 10.0 kg/h, the water flow rate was 15 L/h, and the moisture content was 60% (*w*/*w*). The screw speed was set to 280 rpm. Temperature profiles were established at 50, 70, 110, 140, 145, 150, 150, 140, and 120 °C for sections 2–10, respectively. During the process, the cooling zone was maintained at a temperature below 50 °C via the circulation of water. Subsequently, MA samples were sliced into strips measuring 15 cm in width and 25 cm in length. These MA strips were then preserved in a −20 °C refrigerator for future analytical purposes. A schematic diagram of the extruder is shown in Figure 1.

### 2.3. Texture Properties’ Measurement

The texture distribution of the MA samples was detected using a Brookfield CT3 texture analyzer (Brookfield, WI, USA). The MA samples were cut into rectangular specimens measuring 15 mm in length, 15 mm in width, and 10 mm in height. These specimens were then doubly compressed to 50% of the original height under the texture profile analysis (TPA) mode. The descending and ascending speeds of the probe were set at 2 mm/s, and the testing speed at 0.5 mm/s. Furthermore, the MAs were cut into rectangular samples. The samples were then reduced to 75% of their original thickness at a speed of 1.5 mm/s, both vertically and parallel to the extrusion direction. The lengthwise shear force (FL) and crosswise shear force (FC) were defined as the maximum forces required to cut the sample in the vertical and parallel directions, respectively. The fiber degree (FD) was shown as the ratio of FL to FC.

### 2.4. Color Measurement

An NR60CP+ handheld colorimeter, manufactured by Shenzhen Sanenshi Technology Co., Ltd. in Shenzhen, China, was utilized to monitor the color alterations of the MAs. The device was used to record L* (brightness), a* (redness), and b* (yellowness) values. Subsequently, these values were contrasted with the initial readings to ascertain the magnitude of color alteration. The lightness (L_0_*), redness (a_0_*), and yellowness (b_0_*) of the standard white plate were 97.85, −0.01, and 1.43, respectively. ΔE is given by the following formula:

(1) $$\Delta E=\sqrt{\Delta L^{2}+\Delta a^{2}+\Delta b^{2}}$$

where Δ*L*, Δ*a*, and Δ*b* are the differences between samples and the standard white plate in terms of L_0_*, a_0_*, and b_0_* values, respectively.

### 2.5. SEM Observation

Based on our previous method [21], the microstructure of the sample was observed by SEM (Hitachi Manufacturing Co., Ltd. in Tokyo, Japan). MA samples were freeze-dried and ground into powder using a mortar. Before SEM observation, all samples were sprayed with gold. The magnification was 300 and 1500 times.

### 2.6. Rheological Properties’ Measurement

We refined our existing methodology to determine the rheological properties of the samples [22]. Firstly, the MA freeze-dried sample was ground into powder, and deionized water was added to prepare a 15% (*w*/*v*) solution. After magnetic stirring for 2 h, it was stored at 4 °C overnight. Then, an MCR 101 rheometer (Anton Paar, Graz, Austria) was used to measure it. The linear viscoelastic region was determined by stress scanning prior to measurement. As well as the shear stress and shear rate, apparent viscosity was also measured.

### 2.7. DSC Measurement

The thermal properties of the MA samples were recorded using a DSC-25 differential scanning calorimeter (TA Instrument, New Castle, DE, USA), following methods employed in previous studies [23]. Specifically, 5 mg of the MA sample was accurately weighed, placed into a crucible, and lightly pressed with a mold to ensure that the sample was sealed in the plate. Nitrogen was selected as the protective gas, and the experiment was conducted with a starting temperature of 20 °C, an ending temperature of 170 °C, and a heating rate of 15 °C/min.

### 2.8. Low-Field Nuclear Magnetic Resonance (LF-NMR) Measurement

The moisture distribution of MA samples was analyzed using an NMI20-030V-I LF-NMR analyzer (Niumag Inc., Shanghai, China). The equipment was fitted with a permanent magnet (the sample cavity was placed vertically), the magnetic field intensity was 0.5 ± 0.05 T, the RF pulse frequency range was 2~30 MHz, the RF frequency control accuracy was ≤0.1Hz, the RF pulse accuracy was ≤100 ns, and the maximum sampling bandwidth was ≥2000 KHz. The MA sample was placed at the bottom of the special glass tube for nuclear magnetic resonance, and then inserted into the center of the LF-NMR analyzer, and the spin–spin relaxation time of fresh extrudates was studied. The waiting time was 4000 ms, the echo time was 0.10 ms, and scanning was conducted 6 times. The measured relaxation time was converted into the corresponding relaxation signal components.

### 2.9. Free Sulfhydryl (FS) and Disulfide Bonds (DBs)

An enhanced version of our prior technique was adopted to assess the rheological properties [24]. The DTNB method was utilized to measure the FS quantity of the MA sample. The MA sample was mixed with dH2O, diluted with buffer solution (Tris-Gly Urea), and then DTNB was added. Here is the FS calculation formula:

(2) $$FS\left(\frac{\mu mol}{g}\right)=\frac{73.53\times D\times A_{412}}{C}$$

where 73.53, *D*, *A*_412_, and C respectively represent the DTNB extinction coefficient, dilution coefficient, sample absorbency, and MA concentration (mg/mL).

Tris-Gly urea buffer (15 mL) was added to the MA sample, and it was mixed with β-mercaptoethanol (0.3 mL) and reacted at room temperature for 1 h. Meanwhile, trichloroacetic acid (TCA) was added. After centrifugal precipitation, the precipitate was dissolved in Tris-Gly urea buffer (20 mL), and DTNB (0.2 mL) was added to react for 20 min. The formulas for calculating the total sulfhydryl (*TS*) content and DB content are as follows:

(3) $$TS\left(\frac{\mu mol}{g}\right)=\frac{73.53\times A_{412}}{C}$$

(4) $$\begin{array}{c} DB\left(\frac{\mu mol}{g}\right)=\frac{TS-FS}{2} \end{array}$$

where 73.53, *A*_412_, and *C* respectively represent the *DTNB* extinction coefficient, sample absorbency, and MA concentration (mg/mL).

### 2.10. Statistical Analysis

The samples underwent three replicate tests, and the resulting data were summarized using mean values accompanied by standard deviations. Statistical analysis of the experimental data was conducted using ANOVA and the Tukey test in SPSS 21 software (Chicago, IL, USA), with statistical significance set at *p* < 0.05.

## 3. Results and Discussion

### 3.1. Texture Properties’ Analysis

The texture properties were one of the most important indices used to evaluate the similarity between MAs and animal meat [25]. By testing MA texture properties, such as hardness, springiness, chewiness, and fibrosity, the overall quality of the product could be assessed [26]. This would provide a scientific basis for product development and production. The results of texture data are shown in Table 1. The results show that when the ratio of PPI to SM changed from 1:0 to 0:1, the hardness, springiness, and chewiness of MAs increased first and then decreased. When the ratio of PPI to SM was 6:4, the hardness (62.63 ± 2.77 N), springiness (1.01 ± 0.03), and chewiness (4897 ± 206 g) were the highest. By increasing the proportion of SM, the hardness, springiness, and chewiness of MAs decreased gradually. This could be because there may be an interaction between SM and PPI. In a certain range, the addition of SM may help to enhance the structural stability of MAs, thus improving their texture properties. However, when the content of SM is too high, it may destroy the stable structure originally formed by PPI, resulting in a decline in texture characteristics. When an MA consists of SM only, the hardness, elasticity, and chewiness are the lowest. The taste may be too soft, inelastic, or chewy, which may affect consumers’ eating experience and satisfaction. A previous study found that the hardness of beef is about 76 N, and that of pork is about 55 N [27]. When the ratio of PPI to SM is 6:4, its hardness is closest to that of beef and pork, and this is the best preparation technology of MAs.

The degree of fibrosity is one of the most important indexes to evaluate the quality of plant meat, which is determined according to the ratio of crosswise shear force to lengthwise shear force [28]. Generally speaking, the higher the degree of fibrosity, the better the fiber structure of MAs, which means the higher the similarity between MAs and animal meat [12]. As shown in Table 1, when the ratio of PPI to SM is 6:4, the degree of fibrosity reaches its maximum (1.28 ± 0.05). This shows that adding the proper amount of SM in the extrusion process can lead to the formation of longer MA fiber structures along the extrusion direction. Previous studies have also found that compounding two different plant proteins can effectively improve the degree of fibrosity of MAs [29]. When MAs are only composed of SM, the degree of fibrosity is the lowest (0.74 ± 0.02). This may be because too much SM is not conducive to the orientation of protein molecules in the extrusion process, thus reducing the degree of fibrosity. A previous study found that MAs prepared by adding 30% WG to PPI had the highest degree of fibrosity (2.510 + 0.036) [12]. Based on the importance of the degree of fibrosity in MAs, they think that adding 30% WG is the best process to prepare MAs. Similarly, in our research, PPI:SM = 6:4 is the best preparation technology for MAs.

### 3.2. Color Analysis

Color was an important property of MAs, which affected consumers’ acceptance of the products [28]. The color of MAs primarily depended on the reaction that occurred during extrusion [30]. The influence of the PPI and SM ratio on MA color is shown in Table 2. L* represents brightness (black and white), and the higher the L* value, the brighter the color appeared. If the L* value was positive, it indicated that the sample was brighter than the standard plate; if it was negative, it indicated that the sample was darker [31]. After adding SM, the L* value increased slightly, but only the PPI:SM = 6:4 sample was significantly different. The a* represented red–green chromaticity, where a positive value indicated that the sample was redder than the standard, and a negative value signified that it was greener than the standard [32]. Table 2 shows that a* is positive, indicating that MAs are redder than the standard. After adding SM, the value of a* increased slightly, but there was a significant difference only when PPI:SM = 4:6. In Table 2, b* represents the degree of yellow–blue, and a positive value of b* indicates that the color of the sample was yellower compared to the standard, while a negative value signifies that it was bluer. Table 2 shows that after adding SM to PPI, the value of b* increases significantly. This may be because SM is yellower than PPI. After adding SM to PPI, the Δ*E* value increased significantly (*p* < 0.05). However, there was no significant difference in Δ*E* between MA samples containing different proportions of SM (*p* > 0.05). The enhanced color properties might be leveraged in food products where a richer appearance is desirable, potentially increasing consumer appeal.

### 3.3. SEM Analysis

**Figure 2:**
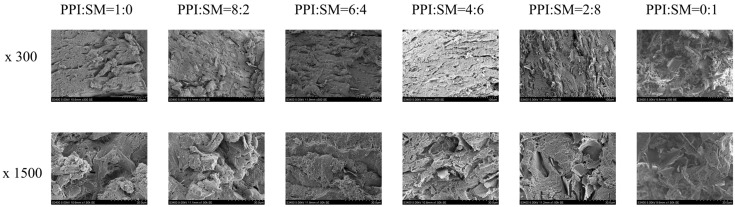
Influence of PPI:SM ratio on MA microscopic image.

Microstructure observation has played a key role in the research on MAs in the past, and the fibrotic structure in MAs can be directly observed via SEM [28]. The microstructures of MAs with different PPI and SM contents were observed under SEM 300 times and 1500 times, as shown in Figure 2. When the ratio of PPI to SM is 1:0, the MA has a few fibrous knots. With the addition of SM, the orientation structure of the MA is more orderly, and the fiber structure is more obvious and detailed along the extrusion direction. When the ratio of PPI to SM is 6:4, the fiber structure in the MA is the most abundant and evenly distributed. This may be because SM and PPI formed two incompatible and independent phases, which prevented protein aggregation and promoted the formation of the fiber structure [33]. With the increase in SM content, the fiber property of the MA decreases. When the ratio of PPI to SM is 0:1, a high level of protein aggregation and dispersion is observed, and the fiber structure of the MA is almost completely destroyed. This may be because excessive SM (PPI:SM ratio is 2:8 and 0:1) interferes with protein recombination, thus damaging the fiber structure of the MA. These changes in microstructure are also reflected in the surface diagram of MA (Figure S1). Previous studies have also found that too much wheat protein will obviously weaken the chewiness of the extrudate, which will hinder the arrangement of protein fibers in the extrusion direction [12]. Although it is not exactly the same as the raw materials used in previous studies, the mechanism of MA fiber structure change may be the same. Generally speaking, when the ratio of PPI to SM is 6:4, MAs contain an obvious fibrous structure, and a small amount of SM can promote the fibrous structure of the MA, while excessive SM will destroy the fibrous structure. This may also be one of the reasons for the decrease in the chewiness and hardness of the MA. The findings suggest that controlling the ratio of PPI to SM is crucial for optimizing the fibrous structure of MAs, which is important for achieving the desirable texture and chewiness in the final product. For industrial applications, this means manufacturers can fine-tune ingredient ratios to produce MAs that closely mimic the texture of real meat, potentially increasing consumer acceptance. Additionally, understanding the interaction between PPI and SM can help in designing efficient extrusion processes, leading to the cost-effective production of high-quality meat alternatives.

### 3.4. Rheological Properties’ Analysis

**Figure 3:**
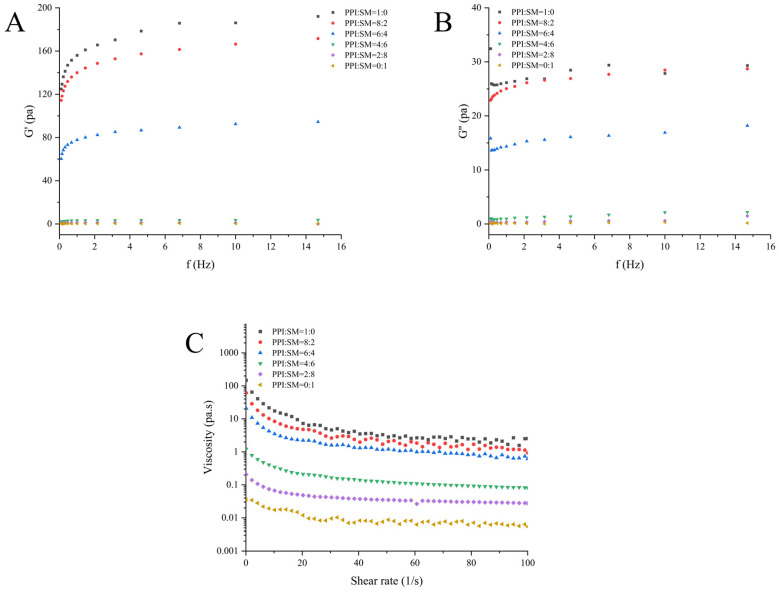
Influence of the PPI:SM ratio on MA rheological properties. (**A**–**C**) respectively represent G′, G″, and the viscosity diagrams of the MA.

Rheological characteristics can reflect the fluidity and viscosity of MAs, as well as the interaction and protein aggregation between different components [28]. The G′ and G″ of MA powder are shown in Figure 3. With the increase in shear frequency, both the G′ and G″ of MA samples increase, and the value of G′ is greater than that of G″. This shows that the elastic properties of MA samples are dominant, indicating that the change in frequency in the linear viscoelastic range will not destroy the structure of an MA. At the same time, it can also be found that with the increase in the SM ratio, both the G′ and G″ of an MA decrease significantly. This may be related to the fact that SM contains more fat, which plays a role in lubrication. This finding is consistent with previous studies, which found that the storage modulus of pea-protein-based MAs decreased significantly with the increase in fat content [34]. Another study on plant-based foods also found that an increase in fat content leads to a decrease in storage modulus and viscosity [35]. Furthermore, the addition of SM may also form protein aggregation pellets with a lubrication function, which can be verified in the SEM images of MAs with a high proportion of SM. Previous studies have also found that adding yeast protein to PPI forms protein aggregate pellets with a lubricating effect, which leads to the decrease in the G′ and G″ of an MA [28].

Figure 3C illustrates the variation in the apparent viscosity of the MA samples as a function of shear rate. As the shear rate rises, the viscosity of the MA sample decreases, demonstrating the sample’s shear-thinning behavior. This is related to the destruction of protein interaction during shearing [26]. In addition, it was found that the viscosity of MAs decreases rapidly at a low-speed shear rate, while it decreases slowly at a high-speed shear rate. This may be due to the destruction of protein clusters in MAs, especially at a high-speed shear rate. At the same shear rate, the viscosity of MAs decreases with the increase in SM content. This may be due to the fact there is more fat in SM, and the lubrication function of protein aggregate pellets. The SEM images of MA samples support this observation.

### 3.5. DSC Analysis

**Figure 4:**
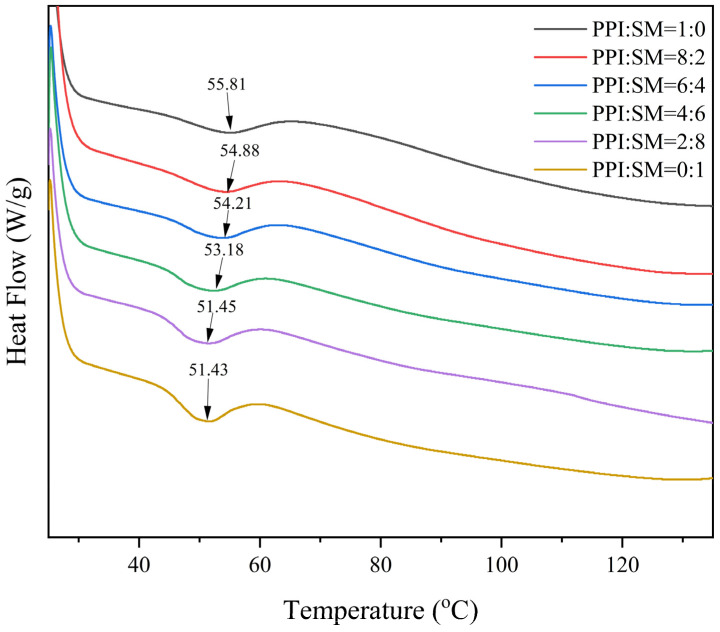
Influence of PPI:SM ratio on DSC thermal profiles.

DSC is a common analytical technique used to measure the degree of protein denaturation [28]. The effects of different proportions of PPI and SM on the thermal stability of MAs are shown in Figure 4. As can be seen from Figure 4, only one endothermic peak was observed on the DSC curve of the MA samples, and the temperature range of thermal denaturation was 51.43–55.81 °C. With the increase in the proportion of SM, the denaturation temperature gradually decreased from 55.81 °C to 51.43 °C. This decrease in denaturation temperature could be due to the interaction between SM and the protein structure of the MA samples, leading to a destabilization of intermolecular bonds. Additionally, the presence of SM might interfere with the protein network, reducing the energy required for denaturation. These results show that SM reduces the thermal stability of MAs, indicating that the molecular structure of MAs with SM can be expanded by lower energy [36]. This discovery shows that adjusting the ratio of PPI to SM can be used to fine-tune the thermal performance of MAs to adapt to specific applications.

### 3.6. LF-NMR Analysis

**Figure 5:**
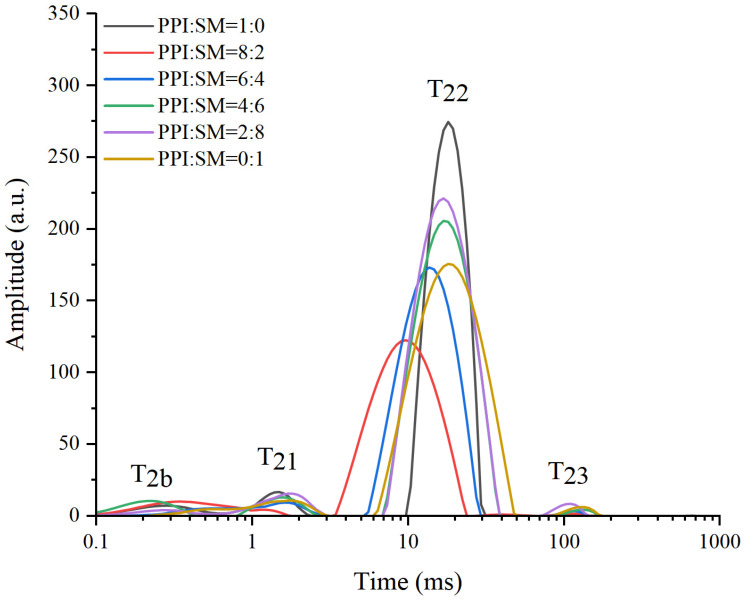
Influence of the PPI:SM ratio on MA T2 relaxation time.

The distribution and migration of water in MAs is an important index, which can be characterized by LF-NMR [28]. For example, the relaxation time distribution curve of the PPI to SM ratio versus MAs is shown in Figure 5. Examining the figure, it becomes evident that as the degrees of freedom increase, the MA peaks can be differentiated into distinct categories: T2b and T21, which represent bound water, T22, corresponding to immobilized water, and T23, signifying free water. Table 3 shows the proportion of T2 distribution, with P22 accounting for the highest proportion, so water molecules mainly exist in the form of fixed water in MAs. With the increase in the proportion of SM, the proportion of P21 in the MA samples showed an upward trend, while P22 and P23 showed a downward trend. The increase in the proportion of bound water may be due to the fact that SM contains more polysaccharides with stronger binding ability to water. Understanding the T2 distribution in MAs is very important for optimizing their structure and hydration properties. By analyzing different forms of water in the sample, researchers can adjust the ratio of PPI to SM to achieve the required moisture content and stability.

### 3.7. FS and DB Analysis

FS and DB are the key substances used to maintain protein spatial conformation and endow proteins with specific functions. The effects of different proportions of PPI and SM on the contents of FS and DB in MAs are shown in Table 4. When the ratio of PPI to SM changed from 1:0 to 0:1, with the increase in SM content, FS content first decreased and then increased. When the ratio of PPI to SM is 6:4, the FS content is the lowest (0.015 0.004 μ mol/g). However, the DB content first increased and then decreased with the increase in SM content. When the ratio of PPI to SM is 6:4, the DB content is the highest (0.582 0.004 μ mol/g). The experimental results show that a certain proportion of SM can promote the formation of DB in MAs. This phenomenon is consistent with the degree of fibrosis and SEM results. This shows that an increase in DB content may be one of the reasons for the strengthening of MAs’ fiber structure.

## 4. Conclusions

As an important source of protein, SM is rich in protein, fat, dietary fiber, vitamins, and minerals. MAs produced by a PPI–SM mixture are helpful to improve the dietary structures and health levels of consumers. In summary, the blending of PPI and SM has been demonstrated to be an effective approach for enhancing the textural properties and fibrous structures of MAs. SEM results indicate that the optimal fibrous structure of MAs is achieved at a PPI to SM ratio of 6:4. Rheological data suggest that the incorporation of SM reduces the viscosity and storage modulus of MAs, which may have a positive impact on reducing energy input during processing. Experimental results on water distribution reveal that water molecules in MAs predominantly exist in a bound state. The addition of SM leads to an increase in the content of bound water in MAs, which is advantageous for improving their storage and processing characteristics. The inclusion of SM not only ameliorates the fibrous structure of MAs but also significantly reduces production costs. However, anti-nutritional compounds, such as trypsin inhibitor and phytic acid in SM, may affect the digestibility and quality of MAs. In the future, it is necessary to further study the effects of anti-nutritional compounds and allergenic elements in SM on the taste and quality of MAs.

## Figures and Tables

**Table 1:** Effect of the ratio of PPI to MS on the texture properties of MAs.

Samples	Hardness (N)	Springiness (mm)	Chewiness (g)	Degree of Fibrosity
PPI:SM = 1:0	44.54 ± 1.69 ^b^	0.80 ± 0.02 ^c^	3173 ± 78 ^c^	0.92 ± 0.03 ^c^
PPI:SM = 8:2	46.71 ± 4.65 ^b^	0.96 ± 0.05 ^a^	3721 ± 146 ^b^	1.15 ± 0.06 ^b^
PPI:SM = 6:4	62.63 ± 2.77 ^a^	1.01 ± 0.03 ^a^	4897 ± 206 ^a^	1.28 ± 0.05 ^a^
PPI:SM = 4:6	38.36 ± 3.07 ^c^	0.85 ± 0.02 ^b^	2728 ± 186 ^d^	0.93 ± 0.05 ^c^
PPI:SM = 2:8	36.03 ± 4.80 ^c^	0.87 ± 0.03 ^b^	2457 ± 353 ^d^	0.85 ± 0.04 ^c^
PPI:SM = 0:1	11.27 ± 1.40 ^d^	0.73 ± 0.02 ^d^	573 ± 90 ^e^	0.74 ± 0.02 ^d^

Note: The results are presented as the mean value along with its standard deviation. Significant differences (*p* < 0.05) are indicated by distinct lowercase letters within the same column.

**Table 2:** Effect of the ratio of PPI to MS on the color values of MAs.

Samples	L*	a*	b*	ΔE
PPI:SM = 1:0	61.76 ± 2.03 ^b^	6.18 ± 1.04 ^b^	22.94 ± 1.00 ^d^	9.53 ± 0.15 ^b^
PPI:SM = 8:2	64.92 ± 4.96 ^ab^	6.61 ± 1.48 ^ab^	27.01 ± 2.27 ^c^	9.92 ± 0.13 ^a^
PPI:SM = 6:4	68.08 ± 2.33 ^a^	6.38 ± 0.98 ^ab^	28.91 ± 2.54 ^bc^	10.16 ± 0.21 ^a^
PPI:SM = 4:6	66.92 ± 6.22 ^ab^	7.93 ± 1.69 ^a^	33.60 ± 4.26 ^a^	10.40 ± 0.31 ^a^
PPI:SM = 2:8	65.60 ± 2.05 ^ab^	7.46 ± 0.41 ^ab^	32.43 ± 2.29 ^ab^	10.27 ± 0.24 ^a^
PPI:SM = 0:1	65.54 ± 2.08 ^ab^	6.81 ± 1.05 ^ab^	32.79 ± 3.45 ^a^	10.25 ± 0.27 ^a^

Note: The results are presented as the mean value along with its standard deviation. Significant differences (*p* < 0.05) are indicated by distinct lowercase letters within the same column.

**Table 3:** Influence of the PPI:SM ratio on MA T2 relaxation time.

Samples	P_21_ (%)	P_22_ (%)	P_23_ (%)
PPI:SM = 1:0	5.945 ± 0.43 ^d^	93.457 ± 0.87 ^a^	0.598 ± 0.02 ^a^
PPI:SM = 8:2	5.252 ± 0.35 ^d^	94.37 ± 1.21 ^a^	0.378 ± 0.03 ^b^
PPI:SM = 6:4	6.51 ± 0.23 ^c^	93.231 ± 1.11 ^a^	0.259 ± 0.04 ^c^
PPI:SM = 4:6	9.725 ± 0.11 ^a^	90.021 ± 1.15 ^b^	0.254 ± 0.06 ^c^
PPI:SM = 2:8	9.088 ± 0.13 ^b^	90.855 ± 0.73 ^b^	0.057 ± 0.01 ^d^
PPI:SM = 0:1	9.151 ± 0.09 ^b^	90.825 ± 0.45 ^b^	0.024 ± 0.01 ^e^

Note: The results are presented as the mean value along with its standard deviation. Significant differences (*p* < 0.05) are indicated by distinct lowercase letters within the same column.

**Table 4:** Effect of the PPI and SM compound ratio on the free sulfhydryl and disulfide bonds of MAs.

Sample	Free Sulfhydryl (μmol/g)	Total Sulfhydryl (μmol/g)	Disulfide Bonds (μmol/g)
PPI:SM = 1:0	0.032 ± 0.004 ^a^	1.079 ± 0.005 ^c^	0.533 ± 0.004 ^d^
PPI:SM = 8:2	0.024 ± 0.005 ^ab^	1.146 ± 0.04 ^a^	0.556 ± 0.008 ^c^
PPI:SM = 6:4	0.015 ± 0.004 ^b^	1.155 ± 0.007 ^a^	0.582 ± 0.004 ^a^
PPI:SM = 4:6	0.025 ± 0.007 ^ab^	1.123 ± 0.003 ^b^	0.549 ± 0.007 ^c^
PPI:SM = 2:8	0.035 ± 0.005 ^a^	1.129 ± 0.007 ^b^	0.569 ± 0.002 ^b^
PPI:SM = 0:1	0.035 ± 0.004 ^a^	1.077 ± 0.008 ^c^	0.511 ± 0.005 ^e^

Note: The results are presented as the mean value along with its standard deviation. Significant differences (*p* < 0.05) are indicated by distinct lowercase letters within the same column.

## Data Availability

The original contributions presented in the study are included in the article/Supplementary Material, further inquiries can be directed to the corresponding author.

## Supplementary Materials

The following supporting information can be downloaded at: https://www.mdpi.com/article/10.3390/foods13233818/s1, Figure S1: The influence of PPI:SM ratio on the surface diagram of MA.
