# Supplementary figures and images for: Effect of Pea Protein Isolate–Soybean Meal Ratio on Fiber Structure and Texture Properties of High-Moisture Meat Analogs
Source: Foods. 2024 Nov 27;13(23):3818. doi: 10.3390/foods13233818 (PMC11640150; doi:10.3390/foods13233818)

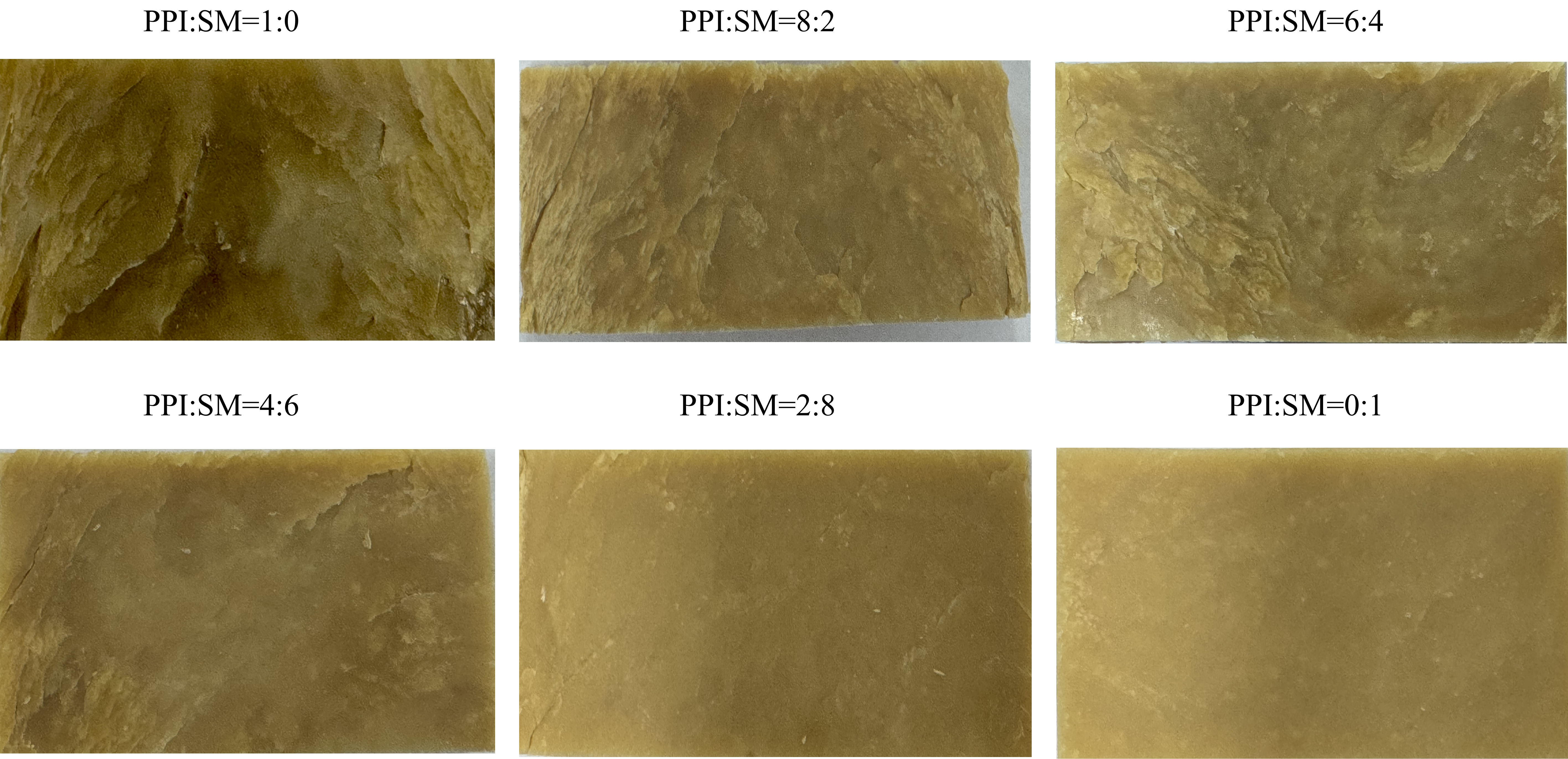

Supplement: Supplementary file 1 [file foods-13-03818-s001.zip › foods-3318242-supplementary.tif]
